# Deubiquitinating enzyme USP10 promotes hepatocellular carcinoma metastasis through deubiquitinating and stabilizing Smad4 protein

**DOI:** 10.1002/1878-0261.12596

**Published:** 2019-11-27

**Authors:** Tao Yuan, Zibo Chen, Fangjie Yan, Meijia Qian, Hong Luo, Song Ye, Ji Cao, Meidan Ying, Xiaoyang Dai, Renhua Gai, Bo Yang, Qiaojun He, Hong Zhu

**Affiliations:** ^1^ Zhejiang Province Key Laboratory of Anti‐Cancer Drug Research College of Pharmaceutical Sciences Zhejiang University Hangzhou China; ^2^ Cancer Hospital of University of Chinese Academy of Sciences, Zhejiang Cancer Hospital Hangzhou China; ^3^ Department of Hepatobiliary and Pancreatic Surgery the Second Affiliated Hospital Zhejiang University School of Medicine Hangzhou China

**Keywords:** deubiquitinating enzyme, hepatocellular carcinoma, metastasis, Smad4, USP10

## Abstract

Hepatocellular carcinoma (HCC) has emerged as one of the most prevalent life‐threatening cancers, and the high mortality rate is largely due to the metastasis. The sustained activation of Smad4 and transforming growth factor‐β (TGF‐β) is closely associated with advanced HCC metastasis. However, the regulatory mechanism underlying the aberrant activation of Smad4 and TGF‐β pathway remains elusive. In this study, using a functional screen of USPs siRNA library, we identified ubiquitin‐specific proteases USP10 as a deubiquitinating enzyme (DUB) that sustains the protein level of Smad4 and activates TGF‐β signaling. Further analysis showed that USP10 directly interacts with Smad4 and stabilizes it through the cleavage of its proteolytic ubiquitination, thus promoting HCC metastasis. The suppression of USP10 by either shRNAs or catalytic inhibitor Spautin‐1 significantly inhibited the migration of HCC cells, whereas the reconstitution of Smad4 was able to efficiently rescue this defect. Overall, our study not only uncovers the regulatory effect of USP10 on the protein abundance of Smad4, but also indicates that USP10 could be regarded as a potential intervention target for the metastatic HCC in Smad4‐positive patients.

## Graphical abstract

Ubiquitin‐specific protease USP10 was shown to activate TGF‐β signaling and promote the metastasis of advanced HCC by deubiquitinating and stabilizing Smad4. Depletion of USP10 or inhibition of the USP10 catalytic activity by use of the small‐molecule inhibitor Spautin‐1 significantly repressed HCC metastasis, whereas reconstitution with Smad4 could efficiently rescue the metastatic phenotype.

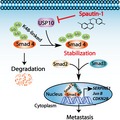

**Abbreviations**
DUB: deubiquitinating enzyme
EMT: epithelial–mesenchymal transition
HCC: hepatocellular carcinoma
rhUSP10: bacterial‐expressed recombinant human USP10
TGFBR I: type I serine/threonine kinase receptors
TGFBR II: type II serine/threonine kinase receptors
TGF‐β: transforming growth factor‐β
USPs: ubiquitin‐specific proteases

## 1 Introduction

Hepatocellular carcinoma (HCC) has emerged as the fifth most frequently diagnosed cancer and the second leading cause of cancer‐related deaths worldwide, especially in South Africa and East Asia (Haider *et al.*, 2019; Kim *et al.*, 2018; Wang *et al.*, 2019). The key reasons for the high mortality rate of HCC are diagnosis at an advanced stage and intrahepatic/extrahepatic metastasis; most of patients often diagnosed at advanced, unresectable, or metastatic HCC; and just a minority of patients is diagnosed at an early stage (Haider *et al.*, 2019; L'Hermitte *et al.*, 2019; Yuan *et al.*, 2014; Zhu *et al.*, 2018). Treatment strategies for HCC remain very limited, and there are no effective therapeutic strategies for metastatic HCC so far (Kim *et al.*, 2018; L'Hermitte *et al.*, 2019). In consideration of the limitations of advanced HCC treatment, novel and effective therapeutic strategies are urgently needed for metastatic HCC.

Multiple studies demonstrated that the overactivation of the transforming growth factor‐β (TGF‐β) pathway is closely associated with advanced HCC metastasis (Bertran *et al.*, 2013; Haider *et al.*, 2019; Reichl *et al.*, 2015; Yuan *et al.*, 2014). TGF‐β is a pleiotropic cytokine that regulates a vast array of biological processes, including proliferation, migration/invasion, and differentiation (Coulouarn *et al.*, 2008; Dupont *et al.*, 2009). TGF‐β exerts its effects through binding type II serine/threonine kinase receptors (TGFBR II) and inducing phosphorylation and activation of type I serine/threonine kinase receptors (TGFBR I). The ligand‐activated TGFBR I then phosphorylates the cytoplasmic transducers, Smad2 and Smad3, which in turn form an active nuclear transcriptional complex with Smad4, to move into nucleus and regulate the transcription of downstream target genes (Coulouarn *et al.*, 2008; Dupont *et al.*, 2009; Haider *et al.*, 2019; Zhang *et al.*, 2017).

Smad4 is a unique and central transducer of TGF‐β responses and is important for most TGF‐β‐induced biological effects, including tumor metastasis/invasion and embryonic development (Dupont *et al.*, 2009). To further define the specific role of Smad4 in HCC development and progression, we analyzed The Cancer Genome Atlas (TCGA) data sets for patients with HCC in order to gain insight on the relevance between Smad4 and HCC pathogenesis. We found that the survival rate significantly decreases in HCC patients with high levels of Smad4 and TGF‐β compared to those with low expression levels (Fig. S1A). Moreover, we found that the mRNA levels of Smad4 closely correlate with the high risk of HCC patients (Fig. S1B) and similar observation was also achieved from that of *SERPINE1/JUNB/CDKN2B*, three well‐known TGF‐β signaling target genes (Fig. S1C). Taken together, these data indicated a close association of Smad4 with HCC progression; thus, the suppression of Smad4 may represent an effective strategy to restrain the metastasis potential of HCC cells.

Given that it is ubiquitination, rather than phosphorylation, which tightly controls the protein abundance and function of Smad4 (Dupont *et al.*, 2009; Liang *et al.*, 2004; Wan *et al.*, 2005; Zhou *et al.*, 2017), we conducted a functional RNA interference screen to globally profile the contribution of ubiquitin‐specific proteases (USPs), the major subfamily of deubiquitinating enzymes (DUBs), to the transcriptional activity of Smad4 in HCC models. Among the several top ‘hits’ which could reinforce this pathway, USP10 stood out since depletion of USP10 using specific shRNA minimizes Smad4 protein levels. Further study demonstrated that USP10 promotes TGF‐β signaling and HCC metastasis through the regulation on the protein abundance of Smad4, but imposing minimal effect on Smad2 and Smad3. Mechanistically, USP10 directly binds to Smad4 and reverts the proteolytic Lys‐48‐linked polyubiquitin chain on Smad4, thus leading to its stabilization. Moreover, ablation of USP10 by either shRNAs or USP10 inhibitor Spautin‐1 significantly suppresses the metastasis of HCC cells *in vitro* and/or *in vivo*, whereas the reconstitution of Smad4 was able to efficiently rescue this defect, indicating that targeting USP10 could be a novel therapeutic strategy of metastatic HCC displaying high level of Smad4.

## 2 Materials and methods

### 2.1 Cell lines and cell culture

The HCC cell lines Bel‐7402 and HepG2 were obtained from Cell Bank of Shanghai Institutes for Biological Sciences, Chinese Academy of Sciences. All the cell lines were authenticated using DNA fingerprinting (variable number of tandem repeats), confirming that no cross‐contamination occurred during this study. Bel‐7402 was maintained in RPMI‐1640 medium (Gibco, Grand Island, NY, USA; #31800) with 10% FBS (Gemini, Woodland, CA, USA; #900‐108), and HepG2 was maintained in Dulbecco’s modified Eagle’s medium (DMEM) (Gibco; #12800) with 10% FBS (ExcellBio, Shanghai, China; #FSP500). All cells were grown at 37 °C in a humidified atmosphere containing 5% CO_2_.

### 2.2 Antibodies and reagents

The antibodies against USP10 (#8501), Smad4 (#38454), Smad2/3 (#3102), β‐TRCP (#4394), vimentin (#3932), and Slug (#3471) were purchased from Cell Signaling Technology, Danvers, MA, USA. N‐cadherin (#610921) was purchased from BD Biosciences, Franklin Lakes, NJ, USA. Anti‐HA‐tag (db2603) and anti‐GAPDH (db106) were purchased from diagbio Biosciences, Hangzhou, China. Anti‐DYKDDDDK‐tag (#A00187), anti‐c‐Myc‐tag (#A00704‐100), and anti‐DYKDDDDK IP resin (#L00425) were purchased from Gen‐Script, Nanjing, China. The anti‐HA affinity gel (#B23302) was purchased from BioTools, Houston, TX, USA. The immunoblotting analysis of proteins in cell lysates was performed as previously described (Zhu *et al.*, 2018). The USP10 inhibitor Spautin‐1 was purchased from TargetMol, Shanghai, China. The Recombinant Human TGF‐β Protein (TGF‐β, 240‐B‐002) was purchased from R&D Systems, Minneapolis, MN, USA.

### 2.3 Gene transfection and real‐time PCR

Plasmids and siRNAs were transfected using Jet PRIME (Polyplus, Strasbourg, France; #114‐15), according to the manufacturer’s instructions, and real‐time PCR was performed as previously described (Zhu *et al.*, 2018). USPs siRNA sequences are listed in Table S1, the sequences of the primer pairs are listed in Table S2, and the relevant shRNAs sequences are listed in Table S3.

### 2.4 Wounding healing assay

Bel‐7402 cells were infected with lentivirus encoding the indicated shRNAs for 16 h and were supplemented with fresh culture medium. After 3 days, these cells were seeded in six‐well dishes at a density of 1 × 10^6^ cells per well and grown to be confluent. The denuded area of constant width was scrapped by using a sterile yellow micropipette tip, and the cell debris was washed with 1× PBS. The wounding closure was monitored and photographed after 24 h.

### 2.5 Transwell migration assay

Transwell migration assays were performed using uncoated polycarbonate membranes with 8 μm pores in 24‐well microchemotaxis chambers. Bel‐7402 cells were infected with lentivirus encoding the indicated shRNAs for 16 h and supplemented with fresh culture medium. After 3 days, 2 × 10^4^ Bel‐7402 cells were seeded into the top of a 24‐well migration chamber in 200 μL serum‐free RPMI 1640, and the lower chamber was filled with 600 μL RPMI 1640 medium containing 10% FBS and 5 ng·mL^−1^ TGF‐β. After incubation for 24 h, the cells migrated to the lower chamber were fixed with 100% ethanol and stained with purple crystal. Then, cells were photographed and counted in three random fields per insert.

### 2.6 Antitumor metastatic activity *in vivo*

BALB/c nude mice were maintained in a pathogen‐free animal facility, and all animal experiments were performed in accordance with the regulations of Institutional Animal Care and Use Committee (IACUC). And the protocols for the animal study were approved by the Animal Research Committee at Zhejiang University, with ethical approval number IACUC‐s19‐006. Every BALB/c nude mice were injected with 1 × 10^6^ cells in 200 μL medium via tail vein. All of the BALB/c nude mice were euthanized 12 weeks postinjection, and liver surface metastatic nodules were enumerated (Hao *et al.*, 2014).

### 2.7 Cell proliferation assay

Bel‐7402 cells were seeded in 96‐well dishes at a density of 1 × 10^4^ cells per well in triplicate and cultured in RPMI 1640 medium containing the indicated concentrations of Spautin‐1 (0, 2.5, 5, and 10 μm) for 24 h, and cell proliferation was assessed by the sulforhodamine B (SRB) assay.

### 2.8 *In vitro* deubiquitination assays

Flag‐tagged Smad4 and HA‐tagged ubiquitin mutant Lys‐K48 only (K48) were transfected into 293T cells. After 36 h, 293T cells were lysed with 4% SDS and then incubated with anti‐DYKDDDDK IP resin overnight at 4 °C. The polyubiquitinated Smad4 from the cell lysate was pulled down by anti‐DYKDDDDK IP resin and incubated with bacterial‐expressed recombinant human USP10 (rhUSP10) protein for 2 h at 37 °C *in vitro*. Subsequently, the ubiquitination levels of Smad4 were analyzed by western blotting.

### 2.9 TGF‐β transcriptional activity assay

The plasmid of TGF‐β transcriptional promoter reporter system (PGL4.14‐SBE_4_‐luc) containing 4‐repeated sequences of ‘GTCTAGAC’ (a sequence of Smad3 and Smad4 specifically recognize and bind to) (Zawel *et al.*, 1998) and a firefly luciferase was utilized to detect the activation of the TGF‐β signaling, and a plasmid containing renilla luciferase was utilized as internal control. These plasmids were transfected into 293T cells that infected with lentivirus encoding the indicated shRNAs. Cells were starved without serum overnight and then incubated with 10 ng·mL^−1^ TGF‐β for 6 h. Subsequently, the luminescence reading obtained with PGL4.14‐SBE_4_‐luc was monitored and divided by the one obtained with renilla luciferase.

### 2.10 USPs siRNA library screening

293T cells were transfected with pooled USPs siRNAs (a mix of oligos for each gene, 50 nm), PGL4.14‐SBE_4_‐luc, and the plasmid containing renilla luciferase and supplemented with fresh culture medium after 5 h. Cells were starved without serum overnight and then incubated with 10 ng·mL^−1^ TGF‐β for 6 h. Subsequently, the luminescence reading obtained with PGL4.14‐SBE_4_‐luc was monitored and divided by the one obtained with renilla luciferase.

### 2.11 Statistical analysis

All experiments were repeated at least three times in our study, and the statistical data were presented as the mean ± SD. Student’s *t*‐test was used to determine statistical significance of the differences, and *P*‐values of 0.05 were considered statistically significant.

## 3 Results

### 3.1 USP10 is a new component of the TGF‐β pathway

**Figure 1:**
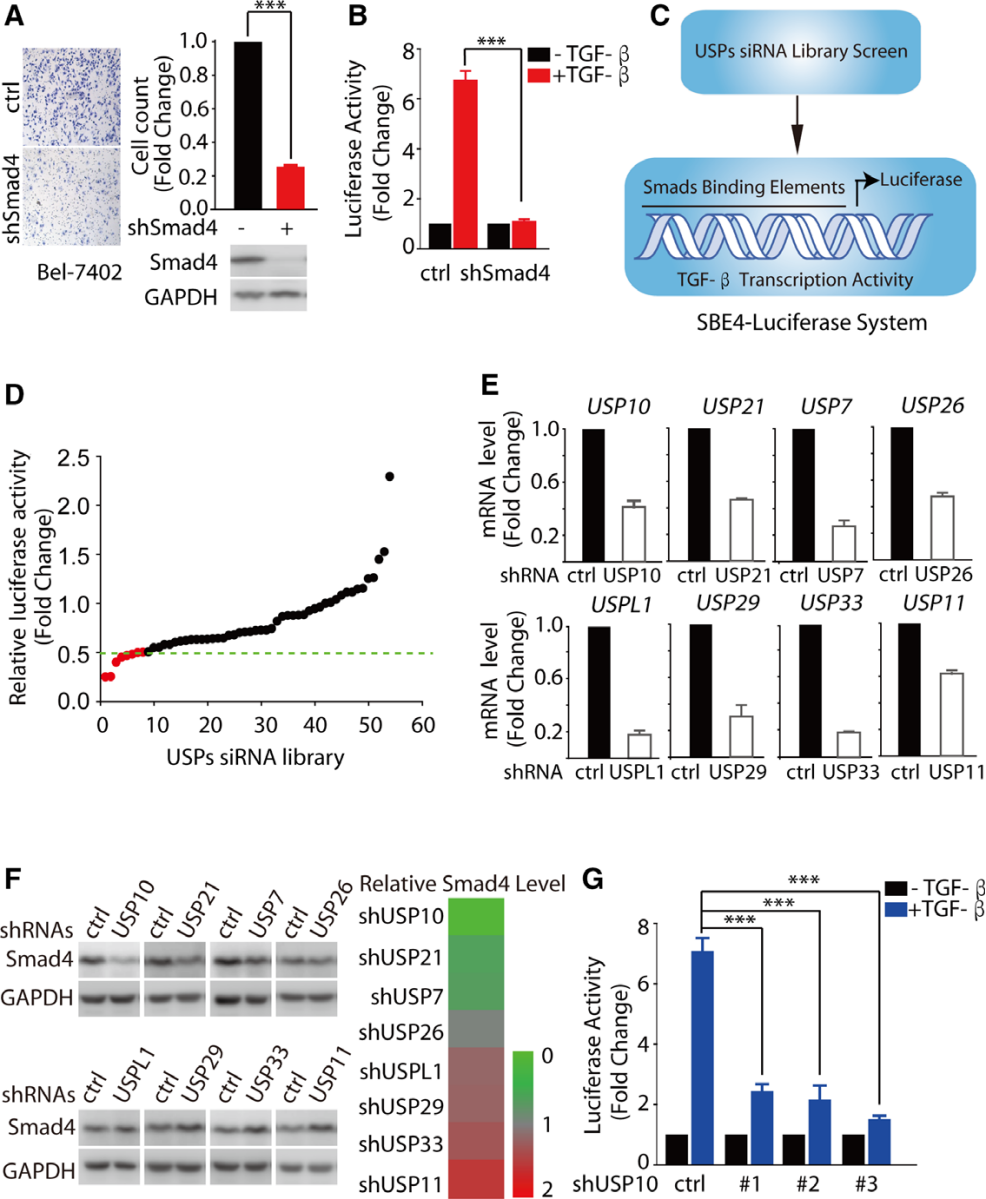
USP10 is a new component of the TGF‐β pathway. (A) Depletion of endogenous Smad4 with shRNAs markedly decreased cell migration by transwell migration assays. Bel‐7402 cells infected with lentivirus encoding the indicated shRNAs were treated with TGF‐β (5 ng·mL^−1^) for 24 h. (B) Depletion of endogenous Smad4 with shRNAs significantly inhibits TGF‐β transcriptional activity. 293T cells infected with lentivirus encoding Smad4 shRNAs were transfected with PGL4.14‐SBE_4_‐luc. Cells were starved without serum overnight and then treated with TGF‐β (10 ng·mL^−1^) for 6 h. (C) A cell‐based luciferase‐screening model to screen the potential USPs regulating TGF‐β signaling by using siRNAs to inhibit the expression of 54 known or predicted human USPs. (D) Eight USPs siRNA pools significantly inhibited TGF‐β responses. Pooled USPs siRNAs (a mix of oligos for each gene, 50 nm) and PGL4.14‐SBE_4_‐luc reporter were transfected in 293T cells, and cells were starved without serum overnight and then treated with TGF‐β (10 ng·mL^−1^) for 6 h. (E, F) Depletion of USP10 minimizes Smad4 protein levels. HepG2 cells were infected with lentivirus encoding the indicated shRNAs. Cell lysates were immunoblotted with indicated antibody and subjected to qRT‐PCR to examine the indicated mRNA levels. Transcript levels were determined relative to *GAPDH* mRNA levels and normalized relative to control cells. (G) The depletion of endogenous USP10 with three independent shRNAs targeting different coding regions of USP10 indeed dramatically inhibits TGF‐β transcriptional activity. 293T cells infected with lentivirus encoding the indicated shRNAs were transfected with PGL4.14‐SBE_4_‐luc. Cells were starved without serum overnight and then treated with TGF‐β (10 ng·mL^−1^) for 6 h. The results represent the means (±SD) of three independent experiments. ***P < 0.001.

Ubiquitin‐specific proteases (USPs) are the largest subfamily of DUBs, and most of them are abnormally activated or expressed in a variety of malignant tumor, making them as ideal anticancer targets (Yuan *et al.*, 2018). Since Smad4, the only co‐Smad of TGF‐β signaling, plays a critical role in the migration and the transcriptional activity of Smad complexes (Fig. 1A–C), we sought to investigate the contribution of USPs involved in Smad4 regulation.

To this end, the cell‐based luciferase‐screening model (Fig. 1C) was utilized to explore the potential USPs regulating TGF‐β signaling by using siRNAs to inhibit the expression of 54 known or predicted human USPs (Table S1). The pooled USPs siRNAs (a mix of oligos for each gene, 50 nm) were transfected in 293T cells. Subsequently, these cells were treated with TGF‐β for 6 h and followed by a TGF‐β transcriptional activity assay. The results showed that siRNA pools for eight USPs including USP7, USP10, USP11, USP21, USP26, USP29, USP33, and USPL1 could remarkably inhibited TGF‐β responses (Fig. 1D, relative luciferase activity < 0.5).

However, among these eight DUBs with potential regulatory effect on TGF‐β transcriptional activity, only the depletion of USP10 could significantly minimize Smad4 protein levels (Fig. 1E,F). The potent inhibitory effect on Smad4 was further verified by the suppression on TGF‐β transcriptional activity, by three independent shRNAs targeting different coding regions of USP10 (Fig. 1G).

Meanwhile, we analyzed The Cancer Genome Atlas (TCGA) data sets for patients with HCC in order to gain insight on the relevance between USP10 and HCC pathogenesis, as shown in Fig. S2A; we found mRNA levels of USP10 closely correlate with the high risk of HCC patients. In line with these data, USP10 influenced the prognosis of HCC patients, as the progression‐free survival (PFS) significantly decreases in hepatitis virus‐related HCC patients harboring higher levels of *USP10* (Menyhart *et al.*, 2018) (http://kmplot.com/analysis/index.php?p =service&cancer=liver_rnaseq and http://kmplot.com/analysis/index.php?p=service&start=1) (Fig. S2B). Moreover, we analyzed Gene Expression Profiling Interactive Analysis (GEPIA) data sets for the gene expression correlation analysis of USP10 and TGF‐β signaling target genes in patients with HCC; we found a positive correlation between *USP10* and *CDKN2B*; and the value of correlation coefficient is 0.49 (Fig. S2C). Taken together, these data show that USP10 is involved in the malignancy of HCC and functions as a novel element in TGF‐β signal transduction.

### 3.2 USP10 positively regulates Smad4 protein levels in HCC

**Figure 2:**
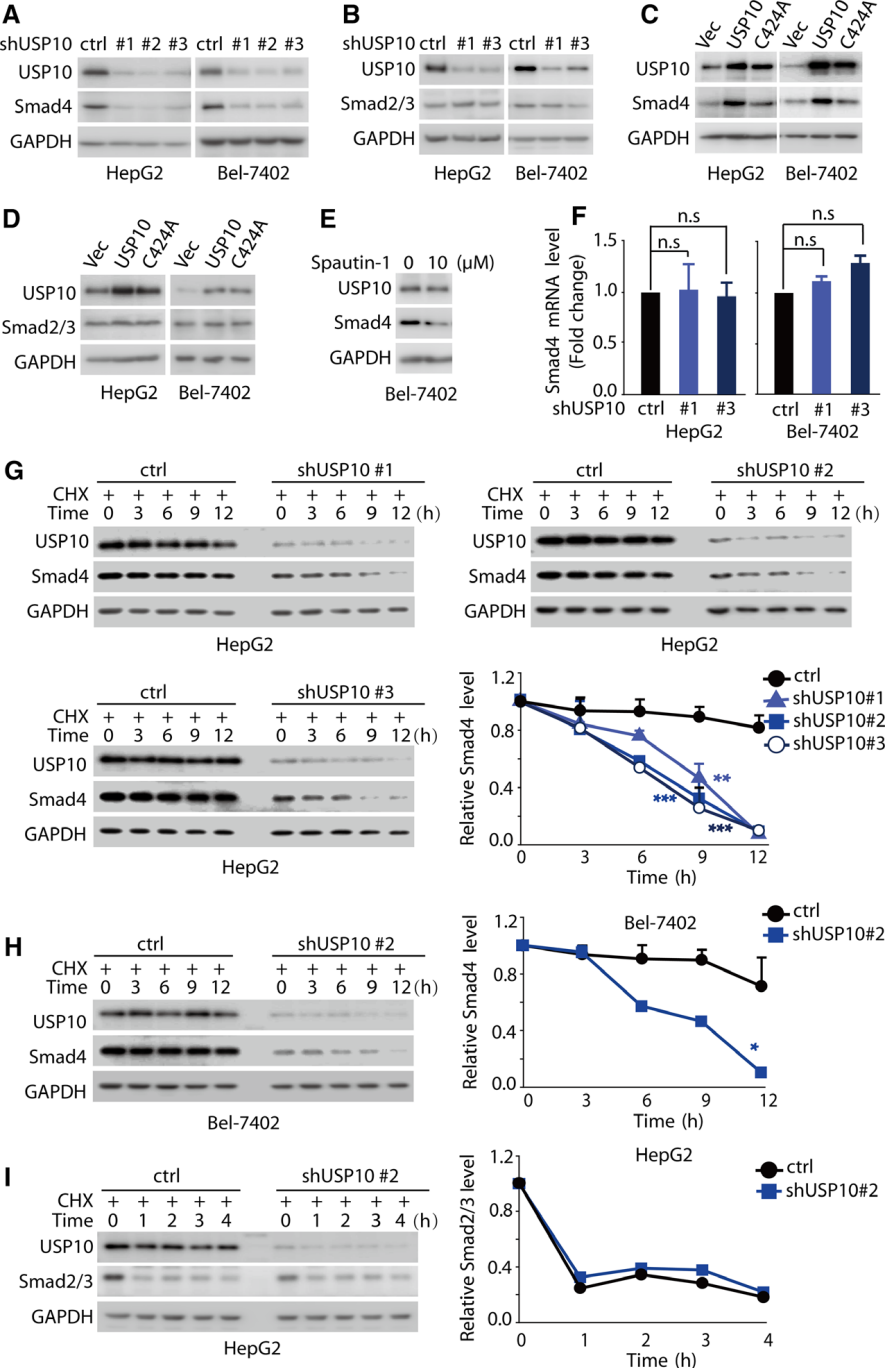
USP10 positively regulates Smad4 protein levels in HCC. (A, B) Knockdown of USP10 down‐regulates Smad4 protein levels, but had little effect on Smad2/3 protein levels. HepG2 and Bel‐7402 cells were infected with lentivirus encoding the indicated shRNAs, and cell lysates were immunoblotted with indicated antibody. (C, D) Overexpression of USP10‐WT, but not USP10‐C424A, up‐regulates the protein levels of Smad4, but not that of Smad2/3. HepG2 and Bel‐7402 cells transfected with indicated constructs were lysed, and cell lysates were immunoblotted with indicated antibody. (E) USP10 inhibitor Spautin‐1 significantly decreased Smad4 protein levels. Bel‐7402 cells were treated for 24 h with 10 μm Spautin‐1, and cell lysates were immunoblotted with indicated antibody. (F) Knockdown of USP10 had little effect on Smad4 mRNA levels. HepG2 and Bel‐7402 cells infected with lentivirus encoding the indicated shRNAs were subjected to qRT‐PCR to examine the indicated mRNA levels. Transcript levels were determined relative to *GAPDH* mRNA levels and normalized relative to control cells. (G, H, I) Knockdown of USP10 dramatically decreased Smad4 protein stability, but not that of Smad2/3. HepG2 and Bel‐7402 cells stably expressing the indicated shRNAs were treated with cycloheximide (CHX, 40 μg·mL^−1^) for the indicated times; then, proteins were extracted and subjected to western blot to examine the indicated protein levels. The results represent the means (±SD) of three independent experiments. ^n.s.^ *P *> 0.05, * *P* < 0.05, **P < 0.01, ***P < 0.001.

The inhibitory effects of USP10 shRNAs on TGF‐β responses led us to examine a potential role of USP10 in the regulation of Smad4 turnover and function. We found that depletion of USP10 using USP10‐specific shRNA in HepG2 and Bel‐7402 cells significantly decreased Smad4 protein levels, but had little effect on Smad2/3 protein levels (Fig. 2A,B and Fig. S3A). However, these inhibitory effects on Smad4 can be restored by the proteasome inhibitor MG132, indicating that USP10 influences Smad4 protein levels through the ubiquitin–proteasome system (Fig. S3B). Conversely, overexpression of USP10‐WT (the wild‐type of USP10) but not USP10‐C424A (the catalytically deficient mutant of USP10) in HepG2 and Bel‐7402 cells dramatically increased the protein levels of Smad4, but not that of Smad2/3 (Fig. 2C,D and Fig. S3C). To further validate the regulatory effect of USP10 on Smad4, we introduced a small‐molecule inhibitor Spautin‐1 (Liu *et al.*, 2011), which can specifically suppress deubiquitinase activity of USP10. As shown in Fig. 2E and Fig. S3D, Spautin‐1 significantly decreased Smad4 protein levels. To demonstrate that the effect of Spautin‐1 on Smad4 is dependent on its catalytic inhibition on USP10, we examined the effect of Spautin‐1 on Smad4 protein levels when depletion of USP10 in HCC cell. As shown in Fig. S3E, when we knocked down USP10 in HCC cell, Spautin‐1 imposed minimal effect on Smad4 protein levels, indicating the effect of Spautin‐1 on Smad4 relies on the existence of USP10. To exclude the possibility that USP10‐induced Smad4 levels were owing to the transcriptional regulation, we performed qRT‐PCR assay to examine mRNA levels of Smad4 in HepG2 and Bel‐7402 cells. The results showed that Smad4 mRNA levels in USP10‐depleted/overexpressed cells remained almost unchanged compared to that of control cells (Fig. 2F and Fig. S3F), suggesting that the effect of USP10 on Smad4 is not mediated through the regulation at the transcriptional levels, but dependent on its deubiquitinase activity.

We then tested whether USP10 can regulate the protein stability of Smad4. HepG2 and Bel‐7402 cells infected with lentivirus encoding USP10 shRNAs were treated with or without the protein synthesis inhibitor cycloheximide (CHX, 40 μm) for the indicated times, and the Smad4 protein levels were analyzed. We found that Smad4 protein was less stable and the half‐life was reduced from > 12 h to 6–9 h upon USP10 depletion (Fig. 2G,H), further denoting that USP10 regulates Smad4 protein levels at the post‐translational level. However, depletion of USP10 imposed little effect on the half‐life of Smad2/3 protein (Fig. 2I and Fig. S3G). Taken together, these results demonstrate that USP10 activates TGF‐β pathway by specifically stabilizing Smad4 protein in HCC models.

### 3.3 USP10 interacts with Smad4 *in vivo*

**Figure 3:**
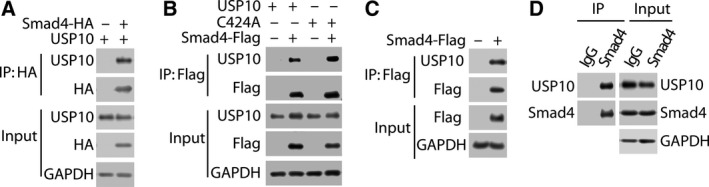
USP10 interacts with Smad4 *in* *vivo.* (A) USP10 interacts with Smad4 *in* *vivo*. 293T cells cotransfected as indicated with expression plasmids encoding HA‐tagged Smad4 and nontagged USP10 cells were lysed, and lysates were incubated with anti‐HA affinity gel. Proteins retained on anti‐HA affinity gel were blotted with the indicated antibodies. (B) The catalytic activity of USP10 is not required for Smad4 binding. 293T cells were cotransfected with expression plasmids encoding Flag‐tagged Smad4 and nontagged USP10 (WT or C424A). Cell lysates were prepared for Co‐IP with anti‐Flag antibody, followed by western blotting as indicated. (C) Endogenous USP10 interacts with Smad4 *in* *vivo*. 293T cells were transfected as indicated with expression plasmids encoding Flag‐tagged Smad4. Cell lysates were immunoprecipitated with anti‐Flag antibody to examine the interaction of Flag‐tagged Smad4 and endogenous USP10. (D) Smad4 interacts with USP10 at endogenous levels. Cell lysates were immunoprecipitated with anti‐Smad4 antibody to examine the interaction of Smad4/USP10 at endogenous levels. The value of Co‐IP/IP ratio is about 15%.

We then explored the mechanism by which USP10 modulates Smad4 stability, firstly by examining whether USP10 is a *bona fide* Smad4 binding protein. The plasmids encoding HA‐tagged Smad4 and nontagged USP10 were co‐expressed in 293T cells, and cells were subsequently harvested for co‐immunoprecipitation (Co‐IP) with the anti‐HA antibody. As shown in Fig. 3A, USP10 could be indeed coprecipitated from cell lysates together with HA‐tagged Smad4 by anti‐HA antibody, suggesting an exogenous interaction between USP10 and Smad4. In addition, 293T cells were cotransfected with plasmids encoding Flag‐tagged Smad4 and nontagged USP10 (WT and C424A mutant, respectively) to examine whether the catalytic activity of USP10 is required for Smad4 binding. As shown in Fig. 3B, USP10‐C424A also efficiently interacted with Smad4 similar as USP10‐WT, suggesting the catalytic activity of USP10 is not required for Smad4 binding. In addition, we also observed an interaction between the Flag‐tagged Smad4 and endogenous USP10 (Fig. 3C). More importantly, the interaction between the endogenous USP10 and endogenous Smad4 was further demonstrated in our Co‐IP analyses (Fig. 3D). Therefore, these results collectively revealed the specific interaction between USP10 and Smad4 both at the endogenous and exogenous levels.

### 3.4 USP10 deubiquitinates and stabilizes Smad4 through the removal of Lys‐48‐linked ubiquitin chains

**Figure 4:**
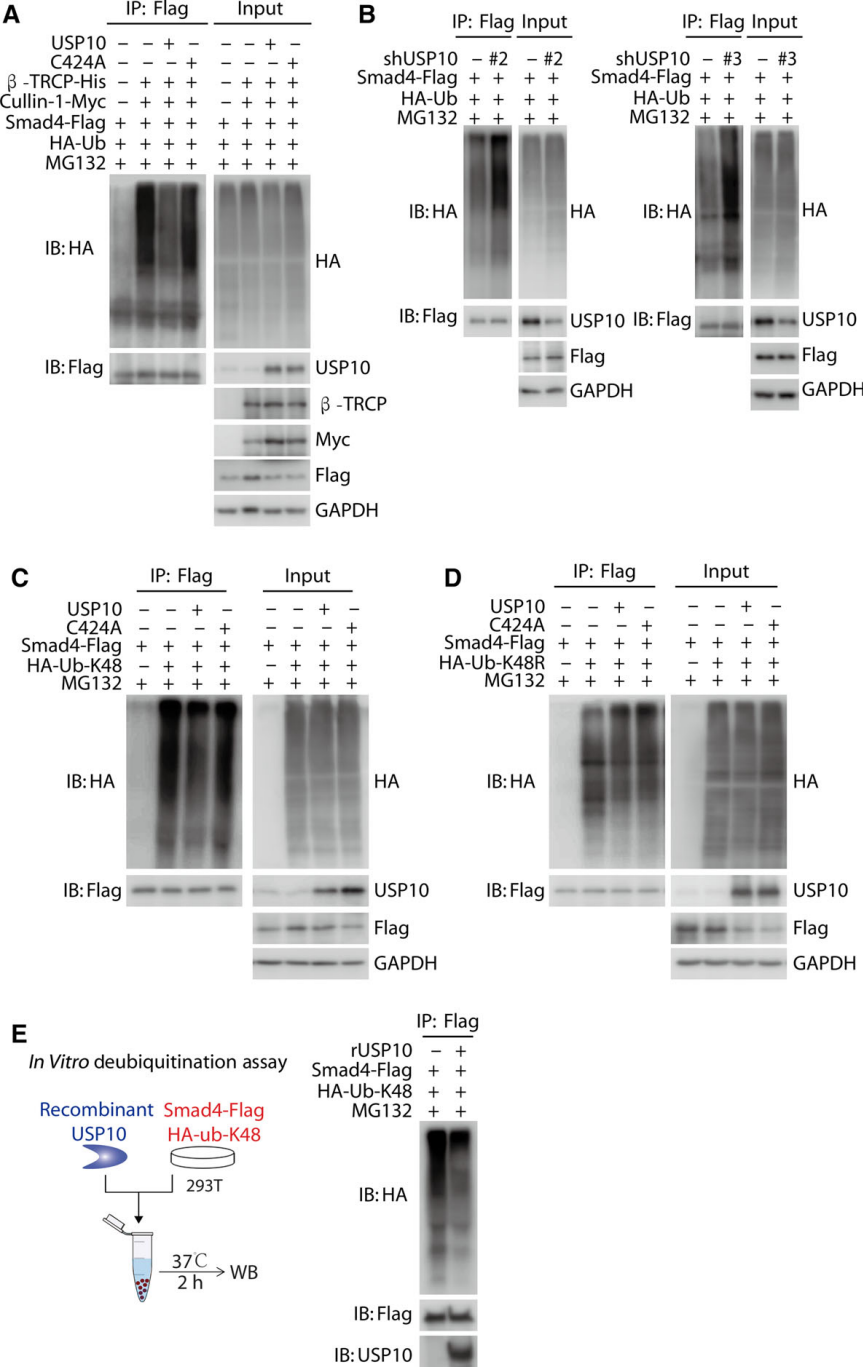
USP10 deubiquitinates and stabilizes Smad4 through the removal of Lys‐48‐linked ubiquitin chains. (A) Co‐expression of USP10‐WT but not USP10‐C424A significantly decreases Smad4 ubiquitination. 293T cells were transfected with expression plasmids encoding for HA‐tagged ubiquitin, Flag‐tagged Smad4, either alone or in combination with His‐tagged β‐TRCP (a known E_3_ Ligase of Smad4), Myc‐tagged cullin‐1, and nontagged USP10 (WT or C424A). Cells were treated with 10 μm MG132 for 8 h before being harvested. Total cell lysates were immunoprecipitated with an anti‐Flag antibody, and the polyubiquitylated Smad4 protein was detected by the anti‐HA antibody. (B) Depletion of USP10 markedly increased Smad4 ubiquitination. 293T cells infected with lentivirus encoding the indicated shRNAs were transfected with HA‐tagged ubiquitin and Flag‐tagged Smad4 and were treated with 10 μm MG132 for 8 h before being harvested. Total cell lysates were immunoprecipitated with an anti‐Flag antibody, and the polyubiquitylated Smad4 protein was detected by the anti‐HA antibody. (C, D) USP10‐WT significantly decreased Lys‐K48‐linked polyubiquitination on Smad4, but imposed little effect on Lys‐K48R‐linked polyubiquitin chains. HA‐tagged ub‐K48 (Lys‐48 only) or ub‐K48R (Lys‐48R) was cotransfected with empty vectors, Flag‐tagged Smad4 and nontagged USP10 (WT or C424A) into 293T cells, which were treated with 10 μm MG132 for 8 h before being harvested. Total cell lysates were immunoprecipitated with anti‐Flag antibody, and the polyubiquitylated Smad4 protein was detected by the anti‐HA antibody. (E) Bacterial‐expressed recombinant human USP10 (rhUSP10) effectively removed the Lys‐K48‐linked polyubiquitination from Smad4 *in vitro*. Flag‐tagged Smad4 and HA‐tagged Lys‐K48‐linked ubiquitin mutant were transfected into 293T cells. Subsequently, polyubiquitinated Smad4 from the cell lysate pulled down by anti‐DYKDDDDK (anti‐Flag) IP resin and incubated with rhUSP10 protein for 2 h at 37 °C *in vitro*. Lysates were immunoblotted with indicated antibodies.

As USP10 directly binds to Smad4 and regulates its protein stability, we hypothesized that USP10 exerts such regulation through the deubiquitination of Smad4. To better illustrate the polyubiquitin chain of Smad4, the proteasome inhibitor MG132 was utilized in the subsequent analyses. Firstly, 293T cells were transfected with expression plasmids encoding for HA‐tagged ubiquitin, Flag‐tagged Smad4, either alone or in combination with His‐tagged β‐TRCP (a known E_3_ Ligase of Smad4, Wan *et al.*, 2005) as well as Myc‐tagged cullin‐1; then, Flag‐tagged Smad4 was immunoprecipitated and its ubiquitination pattern was visualized by immunoblotting against HA‐tagged ubiquitin. As shown in Fig. 4A and Fig. S4A, β‐TRCP dramatically increases the levels of Smad4 ubiquitination. Next, we performed a deubiquitination assay by cotransfecting cells with USP10‐WT or USP10‐C424A, and a significant decrease in polyubiquitylated Smad4 protein observed in cells transfected with USP10‐WT, whereas co‐expression of USP10‐C424A failed to decrease Smad4 ubiquitination (Fig. 4A and Fig. S4A). In contrast, depletion of USP10 markedly increased Smad4 ubiquitination (Fig. 4B and Fig. S4B), indicating that USP10 is an essential DUB for Smad4.

Ubiquitin contains seven lysine (Lys) residues, including Lys‐6, Lys‐11, Lys‐27, Lys‐29, Lys‐33, Lys‐48, and Lys‐63, and every ubiquitin monomer could exert a specific function by forming different polyubiquitin chains (Harrigan *et al.*, 2018; Komander *et al.*, 2009; Yuan *et al.*, 2018). The formation of polyubiquitin chains ultimately determines the destiny of the substrate proteins (Yuan *et al.*, 2018). To further explore which type of polyubiquitin chain of Smad4 was eliminated by USP10, we next examine the types of polyubiquitination occurring on Smad4. We cotransfected expression plasmids encoding for Flag‐tagged Smad4 in combination with HA‐tagged ubiquitin or Lys‐6 only (K6), Lys‐11 only (K11), Lys‐27 only (K27), Lys‐29 only (K29), Lys‐33 only (K33), Lys‐48 only (K48), or Lys‐63 only (K63) ubiquitin mutant. Flag‐tagged Smad4 was immunoprecipitated, and its ubiquitination pattern was visualized by immunoblotting against HA‐tagged ubiquitin. We found that Smad4 had multiple types of polyubiquitination, including the proteolytic Lys‐48‐linked polyubiquitin chains (Fig. S4C).

Aforementioned data suggested that USP10 regulated the protein stability of Smad4, thus raising the possibility that the proteolytic ubiquitination, namely, Lys‐48‐linked polyubiquitin chains (Komander *et al.*, 2009; Yuan *et al.*, 2018), on Smad4, was modulated by USP10. To verify this hypothesis, we asked whether USP10 could cleave the Lys‐K48 (K48)‐only‐ubiquitin chain on Smad4. As shown in Fig. 4C,D, Fig. S4D,E, USP10‐WT significantly decreased Lys‐K48‐linked polyubiquitination on Smad4, but imposed little effect on Lys‐K48R‐linked polyubiquitin chains, indicating that only the Lys‐K48‐linked ubiquitin chains would be removed by USP10. This effect was dependent on the deubiquitination activity of USP10, since the catalytic activity loss mutant USP10‐C424A was incapable to cleave the polyubiquitin chains.

To further confirm the deubiquitination activity of USP10 toward Smad4, we utilized a cell‐free system and performed an *in vitro* deubiquitination assay using bacterial‐expressed recombinant human USP10 (rhUSP10). Flag‐tagged Smad4 and HA‐tagged Lys‐K48‐linked ubiquitin mutant were transfected into 293T cells. Subsequently, polyubiquitinated Smad4 from the cell lysate pulled down by anti‐DYKDDDDK (anti‐Flag) IP resin and incubated with rhUSP10 protein for 2 h at 37 °C *in vitro*. The results showed that rhUSP10 effectively removed the Lys‐K48‐linked polyubiquitination from Smad4 (Fig. 4E and Fig. S4F).

These data collectively demonstrate that USP10 deubiquitinates and stabilizes Smad4 through the cleavage of Lys‐K48‐linked polyubiquitin chain from Smad4, thus preventing its proteasomal degradation.

### 3.5 USP10 depletion inhibits HCC metastasis through down‐regulation of Smad4

**Figure 5:**
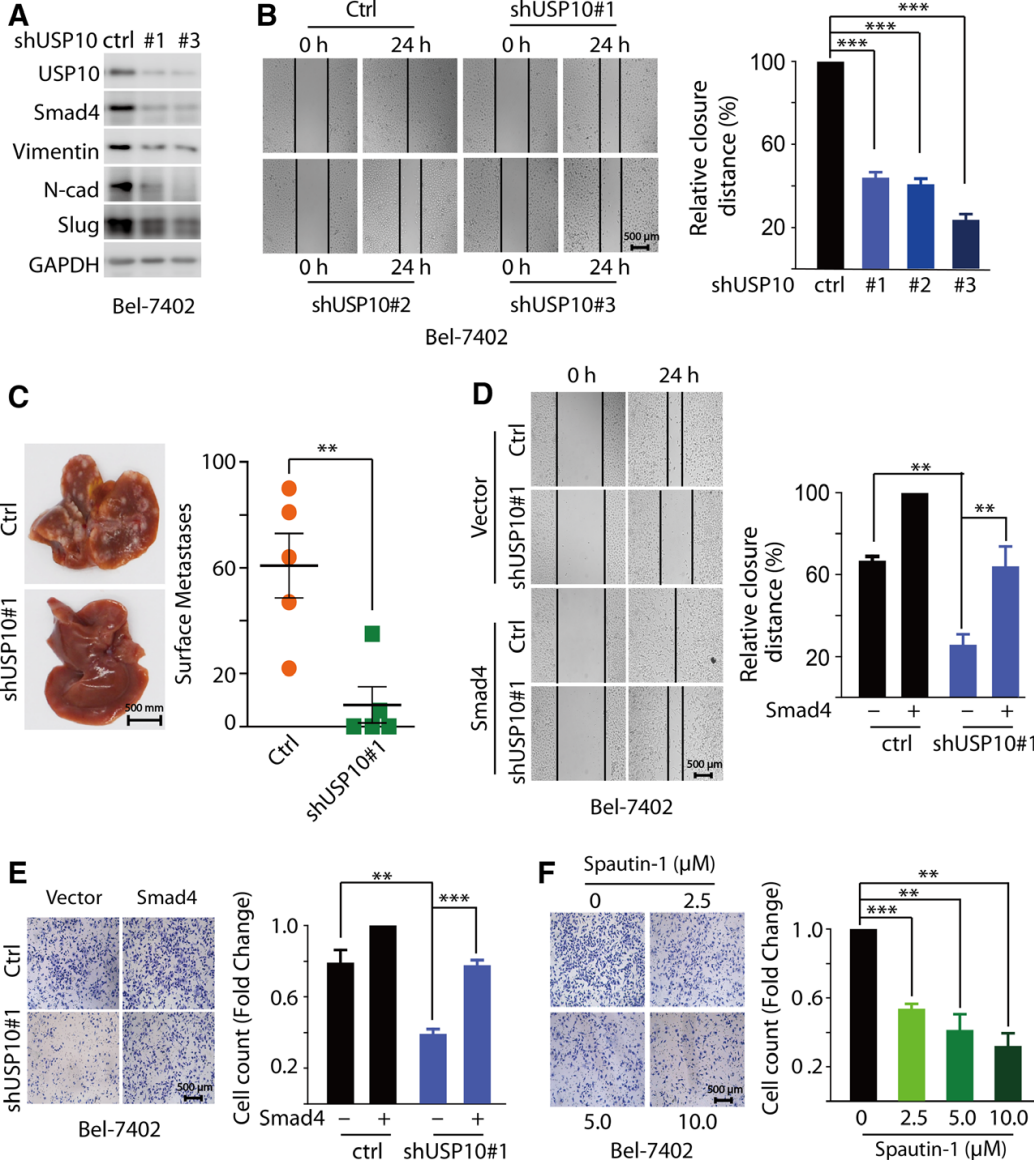
USP10 depletion Inhibits HCC metastasis through down‐regulation of Smad4. (A) Depletion of USP10 in Bel‐7402 cells significantly decreased the expression of EMT marker proteins (N‐cadherin, vimentin, and Slug). Bel‐7402 cells were infected with lentivirus encoding the indicated shRNAs, and cell lysates were immunoblotted with indicated antibody. (B) Depletion of endogenous USP10 with shRNAs markedly decreased cell migration by wounding healing assay. Bel‐7402 cells infected with lentivirus encoding the indicated shRNAs were treated with TGF‐β (5 ng·mL^−1^) for 24 h. (C) Depletion of USP10 in Bel‐7402 cells significantly decreased liver surface metastatic nodules of BALB/c nude mice. A representative image of liver was shown, and liver surface metastatic nodules were enumerated. (D, E) Overexpression Smad4 reversed cell migration by wounding healing assay (D) or transwell migration assays (E). Bel‐7402 cells infected with lentivirus encoding the indicated shRNAs and (or) plasmids were treated with TGF‐β (5 ng·mL^−1^) for 24 h. (F) Spautin‐1 inhibited HCC metastasis in a dose‐dependent manner. Bel‐7402 cells were treated with TGF‐β (5 ng·mL^−1^) and the indicated concentrations of Spautin‐1 (0, 2.5, 5, and 10 μm) for 24 h. The results represent the means (±SD) of three independent experiments. **P < 0.01, ***P < 0.001.

Canonical TGF‐β/Smad4 signaling has pleiotropic functions, among which, growth arrest is the main response induced in normal epithelia or early carcinomas, whereas promotion of invasion and metastasis behaviors prevails in advanced tumors (Coulouarn *et al.*, 2008; Dupont *et al.*, 2009; Reichl *et al.*, 2015). In this context, we examined the function of USP10 in the metastasis of HCC cells. Epithelial–mesenchymal transition (EMT) plays fundamental roles in the early metastatic tumor by endowing tumor cells with a more motile and invasive potential (Yuan *et al.*, 2014), so we firstly examined the effect imposed by USP10 on EMT‐relevant proteins. As shown in Fig. 5A, depletion of USP10 in Bel‐7402 cells significantly decreased the expression of EMT marker proteins (N‐cadherin, vimentin, and Slug). Next, we depicted the roles of USP10 on HCC metastasis using wounding healing assays and migration assays. Figure 5B displays that depletion of endogenous USP10 with shRNAs markedly decreased cell migration compared to that of the control cells, as indicated by the inhibition rate on wound healing as 56.15%, 59.19%, and 76.42%, respectively (*P* < 0.001). Notably, depletion of USP10 imposed minimal effect on the cell proliferation, at least under the experimental conditions (Fig. S5A). To extend our *in vitro* observations concerning USP10’s role in promoting HCC metastasis, we evaluated whether USP10 could promote HCC metastasis *in vivo*. Firstly, we stably knocked down USP10 in Bel‐7402 cells. Subsequently, these cells were intravenously injected into BALB/c nude mice. As shown in Fig. 5C, compared with the parental Bel‐7402 cells, depletion of USP10 in Bel‐7402 cells significantly decreased liver surface metastatic nodules of BALB/c nude mice. Taken together, these data suggested that depletion of USP10 significantly inhibits HCC metastasis both *in vitro* and *in vivo.*

In order to demonstrate that Smad4 indeed involved in the regulation of USP10 promoting HCC metastasis, we investigated whether Smad4 could rescue the metastasis defect caused by USP10 depletion. As shown in Fig. 5D of wounding healing assays, when overexpressed Smad4, the cell motile potential was reversed compared to USP10 depletion. Similar observation was also achieved from the transwell migration assays (Fig. 5E and Fig. S5B).

To further verify the function of USP10 on HCC metastasis, the small‐molecule inhibitor Spautin‐1 was introduced. We found that Spautin‐1 inhibited HCC metastasis in a dose‐dependent manner and the inhibition rate is 46.18% (*P* < 0.001), 58.72% (*P* < 0.01), and 67.95% (*P* < 0.01), for 2.5, 5, and 10 μm, respectively (Fig. 5F). Interesting, Spautin‐1 imposed minimal effect on the cell proliferation (Fig. S5C), suggesting that under our experimental conditions, USP10 mainly modulated cell migration rather than cell proliferation, through the regulatory effect on Smad4. We then examined the protein levels of USP10 and Smad4 in diethylnitrosamine (DEN)‐induced HCC mice. As shown in Fig. S5D, the protein levels of USP10 were more abundant and highly correlated with Smad4 expression in tumorous regions compared with that in nontumorous tissues.

These results demonstrate that USP10 reinforces the TGF‐β signaling and promotes the metastasis of HCC cells. Moreover, depriving of USP10 catalytic activity by small‐molecule inhibitor exerts a significant repression on HCC metastasis.

## 4 Discussion

Metastasis remains an unsolved problem that accounts for the high mortality of HCC patients, and the lack of intervention targets against metastasis greatly hinders the improvement of HCC treatment. The current study initiated a functional screening of USPs siRNA library on the TGF‐β signaling and identified that USP10 acts as a new regulator of Smad4 to reinforce the TGF‐β/Smad4 pathway, thus ultimately leads to the enhanced metastasis of HCC cells. Mechanically, this prometastatic effect of USP10 was exerted through its deubiquitination and stabilization of Smad4 through the cleavage of K48‐linked polyubiquitin chains from Smad4. Furthermore, targeting USP10 by either shRNAs or small‐molecule inhibitor Spautin‐1 evidently inhibits HCC metastasis, revealing that targeting USP10 could be a novel therapeutic strategy against HCC metastasis and the specific inhibitors of USP10 could be potential antimetastatic agents.

Smad4 functions as a vital transcriptional factor of TGF‐β signaling and is indispensable for TGF‐β responses as well as relevant biological effects (Dupont *et al.*, 2009; Ogawa *et al.*, 2019). In view of the pleiotropic functions of canonical TGF‐β signaling, Smad4 is extensively involved in tumor progression, playing drastically different roles in varied tumor models. Several lines of evidence showed that Smad4 may act as a tumor suppressor in colorectal cancer (Ogawa *et al.*, 2019; Wasserman *et al.*, 2018; Yan *et al.*, 2016) and pancreatic cancer (Blackford *et al.*, 2009; Xu *et al.*, 2010). Wasserman and the colleagues demonstrated that Smad4 is closely associated with the high risk of recurrence or death of colorectal cancer patients, and the loss of Smad4 predicts worse clinical outcome, decreased immune infiltrate, and resistance to chemotherapy (Wasserman *et al.*, 2018). In pancreatic cancers, the inactivation of Smad4 significantly correlates with poor prognosis in patients with surgically resected adenocarcinoma of the pancreas (Blackford *et al.*, 2009).

In contrast, Smad4 was also found to play important oncogenic roles in a vast diversity of cancer types, including breast cancer (Taylor *et al.*, 2013; Xue *et al.*, 2014), ovarian cancer (Chan *et al.*, 2017), glioblastoma (Eichhorn *et al.*, 2012), and HCC (Huang *et al.*, 2018; Moon *et al.*, 2017). And metastasis is the most common outcome in TGF‐β‐hyperactivated cancer cells, generally owing to the promotion of epithelial–mesenchymal transition (Bertran *et al.*, 2013; Taylor *et al.*, 2013). Particularly, in HCC models, TGF‐β/Smad4 promotes invasion/metastasis by promoting ERK pathway‐mediated FGFR4 expression (Huang *et al.*, 2018) or through lncRNA activated by TGF‐β (lncRNA‐ATB) by competitively binding the miR‐200 family and up‐regulating ZEB1/2 (Yuan *et al.*, 2014). In line with these studies, we found that the activation of TGF‐β/Smad4 is closely correlated with the poor prognosis of HCC patients. These clues collectively implicate that TGF‐β/Smad4 signaling is fundamental for the malignancy of HCC; consequently, the interruption of TGF‐β/Smad4 signaling is a potential antimetastatic therapy for advanced HCC.

It has been well‐established that phosphorylation of Smad2 and Smad3 dictates their nuclear‐cytoplasmic shuttling, thus plays an essential role in the regulation of these R‐smads (Dupont *et al.*, 2009; Haider *et al.*, 2019; Zhang *et al.*, 2017). On the contrary, for the only known co‐Smad, namely, Smad4, ubiquitination/deubiquitination may represent a more critical mechanism to regulate Smad4, since ubiquitin ligases β‐TRCP and Skp2 have been found to control the abundance of this only known co‐Smad (Izzi and Attisano, 2006; Liang *et al.*, 2004; Wan *et al.*, 2005), whereas minimal impact is imposed by phosphorylation. Several lines of evidence have revealed that USP9X and USP4 removed the mono‐ubiquitination of Smad4 in lysine 519, promoting TGF‐β responses (Dupont *et al.*, 2009; Zhou *et al.*, 2017). However, it still remained to be known which DUB(s) could control the protein stability. In the current study, we found that USP10 imposed an important reinforcing effect on Smad4, by cleaving its proteolytic polyubiquitin chain and the subsequent stabilization.

Several lines of evidence have unraveled the diverse roles of USP10 in tumorigenesis as well as malignancy in different cancer types (Guturi *et al.*, 2016; Lin *et al.*, 2013; Sun *et al.*, 2018; Weisberg *et al.*, 2017; Yang *et al.*, 2014; Yuan *et al.*, 2010). Lin *et al.* found that USP10 antagonizes the transcriptional activity of c‐Myc by deubiquitinating and stabilizing SIRT6, to inhibit cell growth and tumor formation in colon cancers (Lin *et al.*, 2013). Yuan *et al.* showed that USP10 reversed MDM2‐mediated p53 nuclear export and degradation by deubiquitinating and stabilizing cytoplasmic p53 (Yuan *et al.*, 2010). Under DNA damage, USP10 becomes stabilization and translocates to the nucleus to activate and stabilize nuclear p53. Furthermore, USP10 inhibits tumor cell proliferation by increasing p53 levels in cells with wild‐type p53, but exacerbates tumorigenesis in tumor cells with mutant p53 background. However, in acute myeloid leukemia (AML) patients with activating mutations in FMS‐like tyrosine kinase 3 (FLT3), USP10 deubiquitinates and stabilizes FLT3 by removing the proteolytic polyubiquitin chains and preventing FLT3 degradation, to aggravate tumor progress (Weisberg *et al.*, 2017). These studies indicated that the diverse functions of USP10 are largely dictated by its substrates in the specific cell context of the cancer models. Nevertheless, the roles of USP10 in cancer metastasis remain to be clarified. Ouchida *et al.* (2018) identified that USP10 functions as a deubiquitinase and regulator of the EMT‐transcription factor Slug to promote cell migration in multiple tumor cells, including non‐small‐cell lung carcinoma, ovarian cancer, fibrosarcoma, and breast cancer. However, the functions of USP10 on HCC metastasis are still unclear. The current study identified the prometastatic roles of USP10 in HCC and found that USP10 promotes TGF‐β signaling and HCC metastasis by stabilizing Smad4 through the cleavage of proteolytic K48‐linked ubiquitination.

## 5 Conclusions

In summary, our data provide insights into the function of USP10 on HCC metastasis. We found that USP10 plays a key role in the metastasis of advanced HCC by deubiquitinating and stabilizing Smad4, a vital transcriptional factor of TGF‐β signaling. Furthermore, we demonstrate that depletion of USP10 or depriving of its catalytic activity with small‐molecule inhibitor Spautin‐1 significantly represses the metastasis of HCC cells *in vitro* and/or *in vivo*, whereas the reconstitution of Smad4 was able to efficiently rescue this defect. Taken together, our study not only identifies USP10 as a new prometastatic factor in HCC, but also unravels the mechanistic framework by which USP10 reinforces the TGF‐β signaling through the stabilization of Smad4, thus provides potential therapeutic targets for the treatment against metastatic HCC in Smad4‐positive patients.

## Conflict of interest

The authors declare no conflict of interest.

## Author contributions

TY, ZC, BY, QH, and HZ conceived and designed the project; TY, ZC, FY, and MQ acquired the data; TY, ZC, FY, MQ, BY, QH, and HZ analyzed and interpreted the data; TY, ZC, QH, and HZ wrote and revised the manuscript; HL, SY, JC, MY, XD, and RG provided administrative, technical, or material support.

## Supporting information


**Fig. S1.** Positive correlation between Smad4 protein levels and HCC progression.

**Fig. S2.** Positive correlation between USP10 protein and HCC progression.

**Fig. S3.** USP10 regulates Smad4 protein levels through the ubiquitin‐proteasome system.

**Fig. S4.** The types of poly‐ubiquitination occurring on Smad4.

**Fig. S5.** Depletion of USP10 or depriving of its catalytic activity with small molecule inhibitor Spautin‐1 imposed minimal effect on the cell proliferation.

**Table S1.** Complete list of the siRNAs targeting 54 human known or predicted ubiquitin‐specific proteases (USPs).
**Table S2.** The primer sequences for qRT‐PCR assay.
**Table S3.** The targeting sequences for shRNA.
