# Supplementary figures and images for: Deubiquitinating enzyme USP10 promotes hepatocellular carcinoma metastasis through deubiquitinating and stabilizing Smad4 protein
Source: Mol Oncol. 2019 Nov 27;14(1):197–210. doi: 10.1002/1878-0261.12596 (PMC6944132; doi:10.1002/1878-0261.12596)

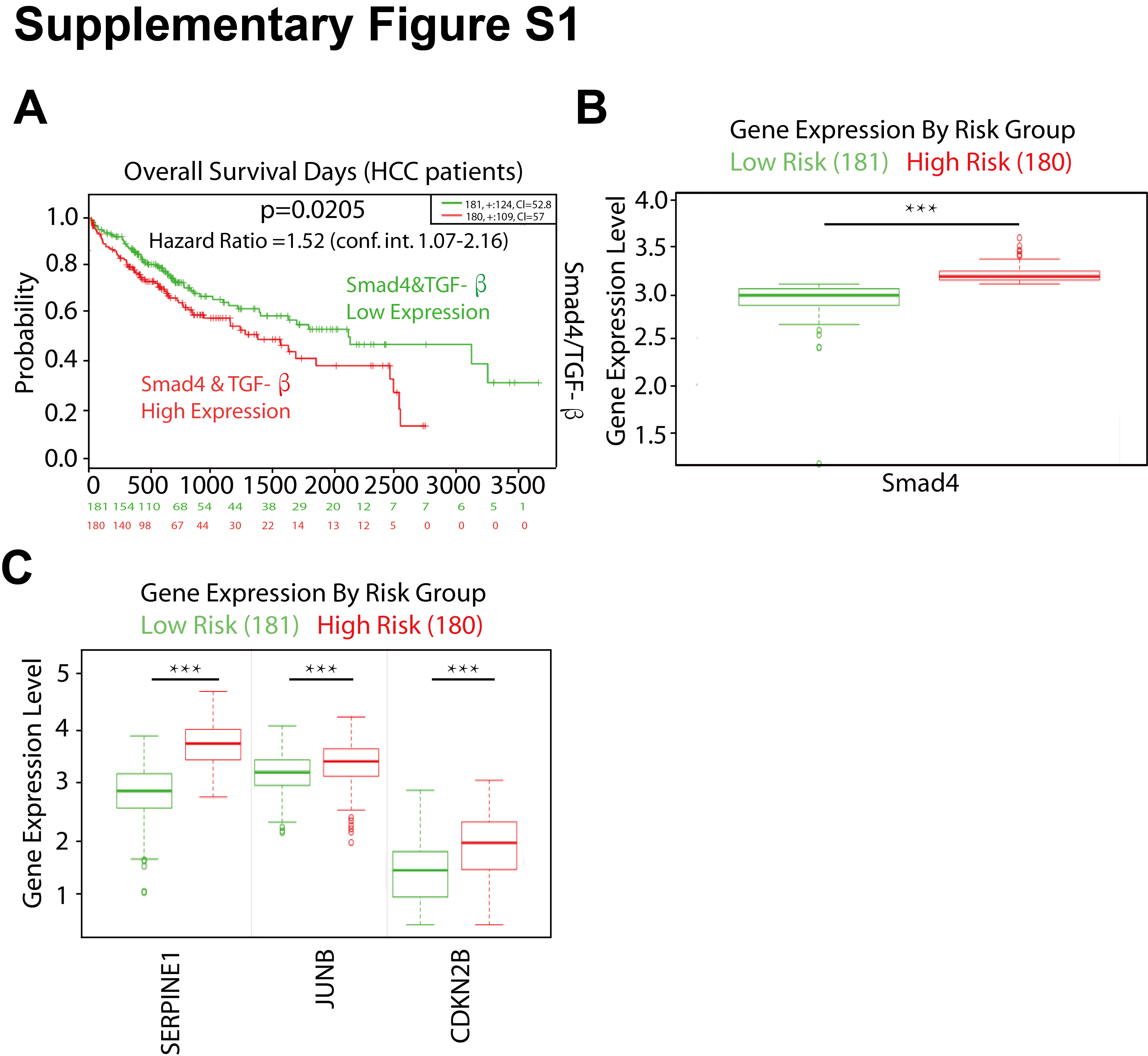

Supplement: Supplementary file 1 — Fig. S1. Positive correlation between Smad4 protein levels and HCC progression. [file MOL2-14-197-s001.tif]

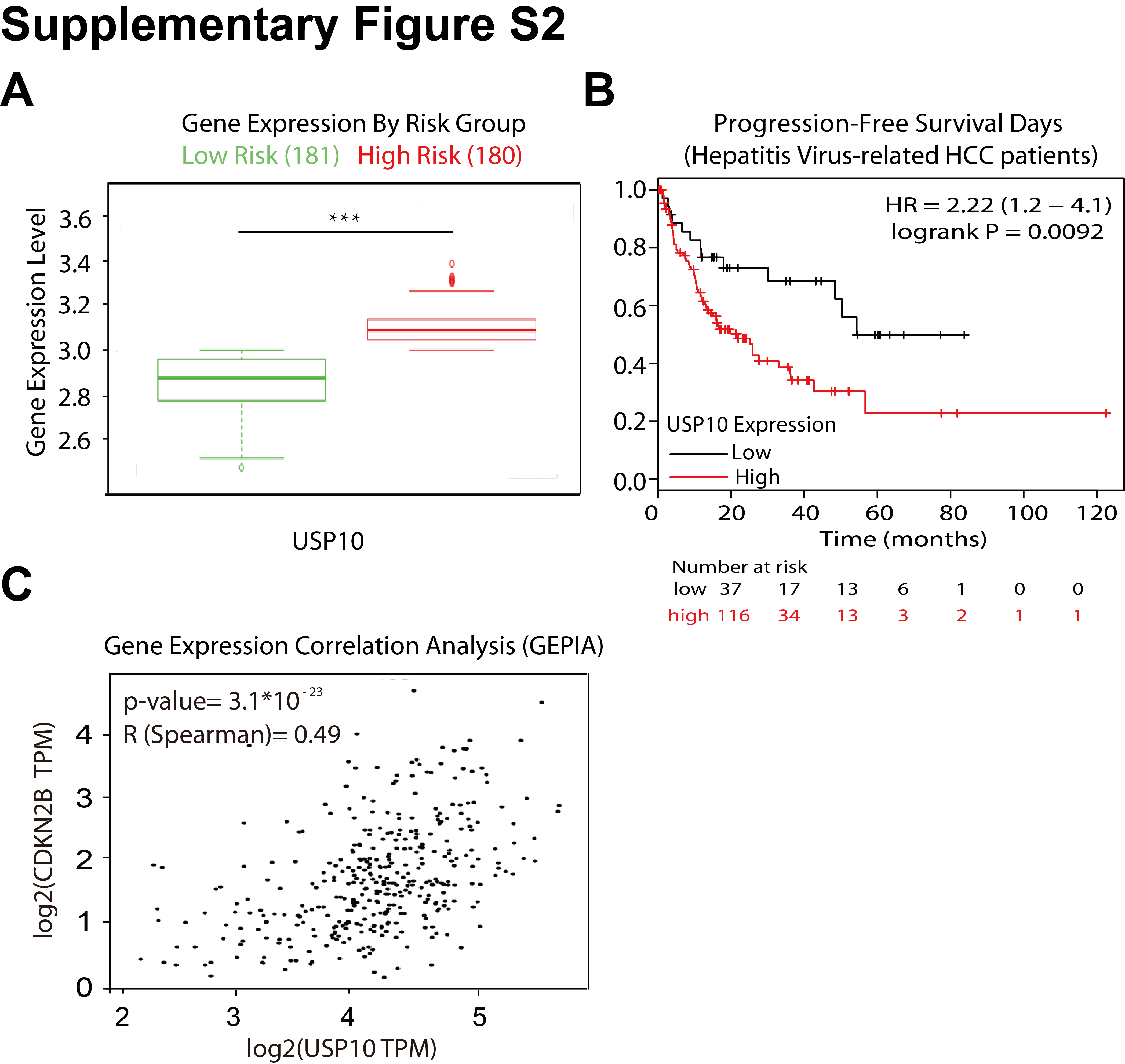

Supplement: Supplementary file 2 — Fig. S2. Positive correlation between USP10 protein and HCC progression. [file MOL2-14-197-s002.tif]

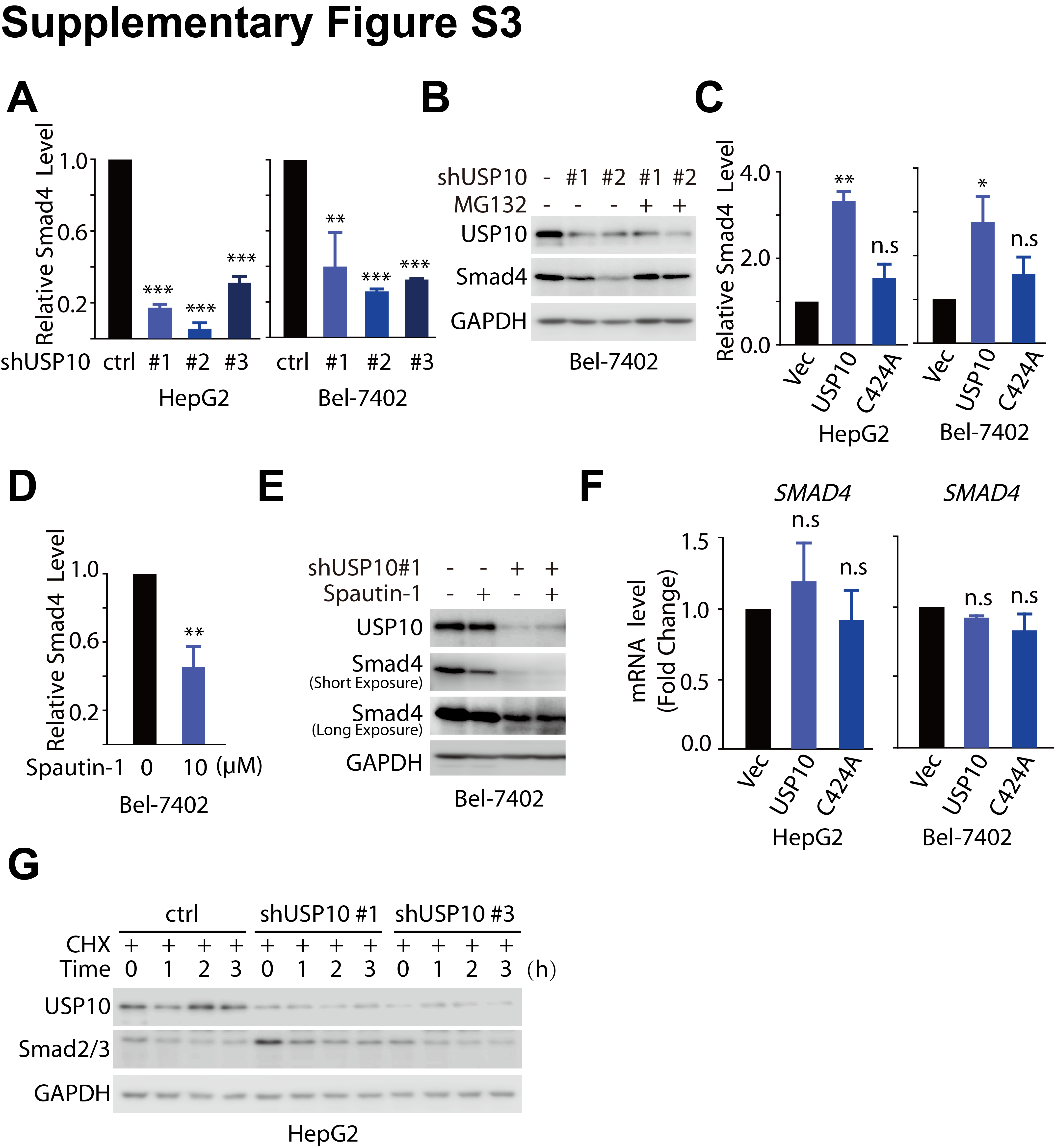

Supplement: Supplementary file 3 — Fig. S3. USP10 regulates Smad4 protein levels through the ubiquitin‐proteasome system. [file MOL2-14-197-s003.tif]

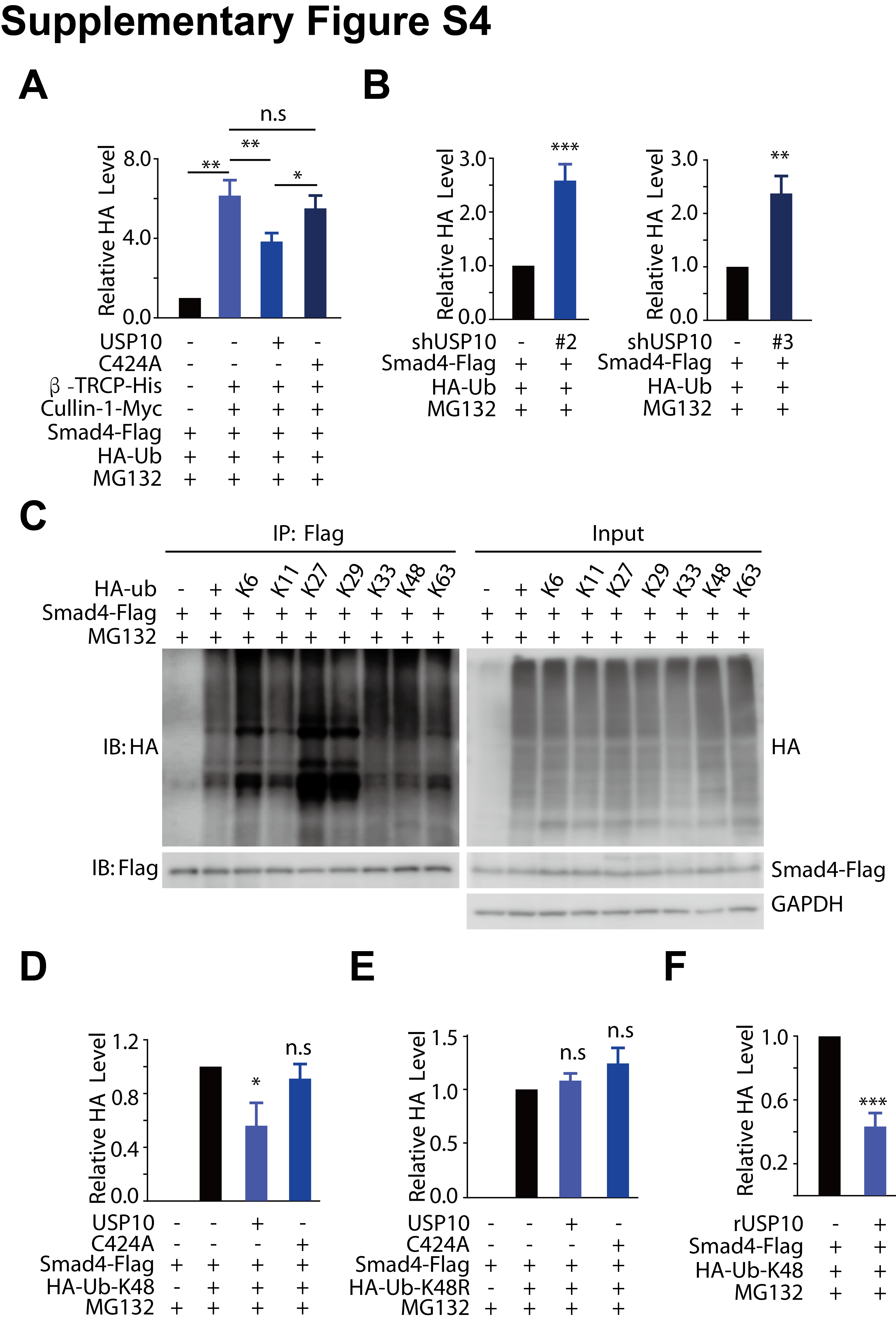

Supplement: Supplementary file 4 — Fig. S4. The types of poly‐ubiquitination occurring on Smad4. [file MOL2-14-197-s004.tif]

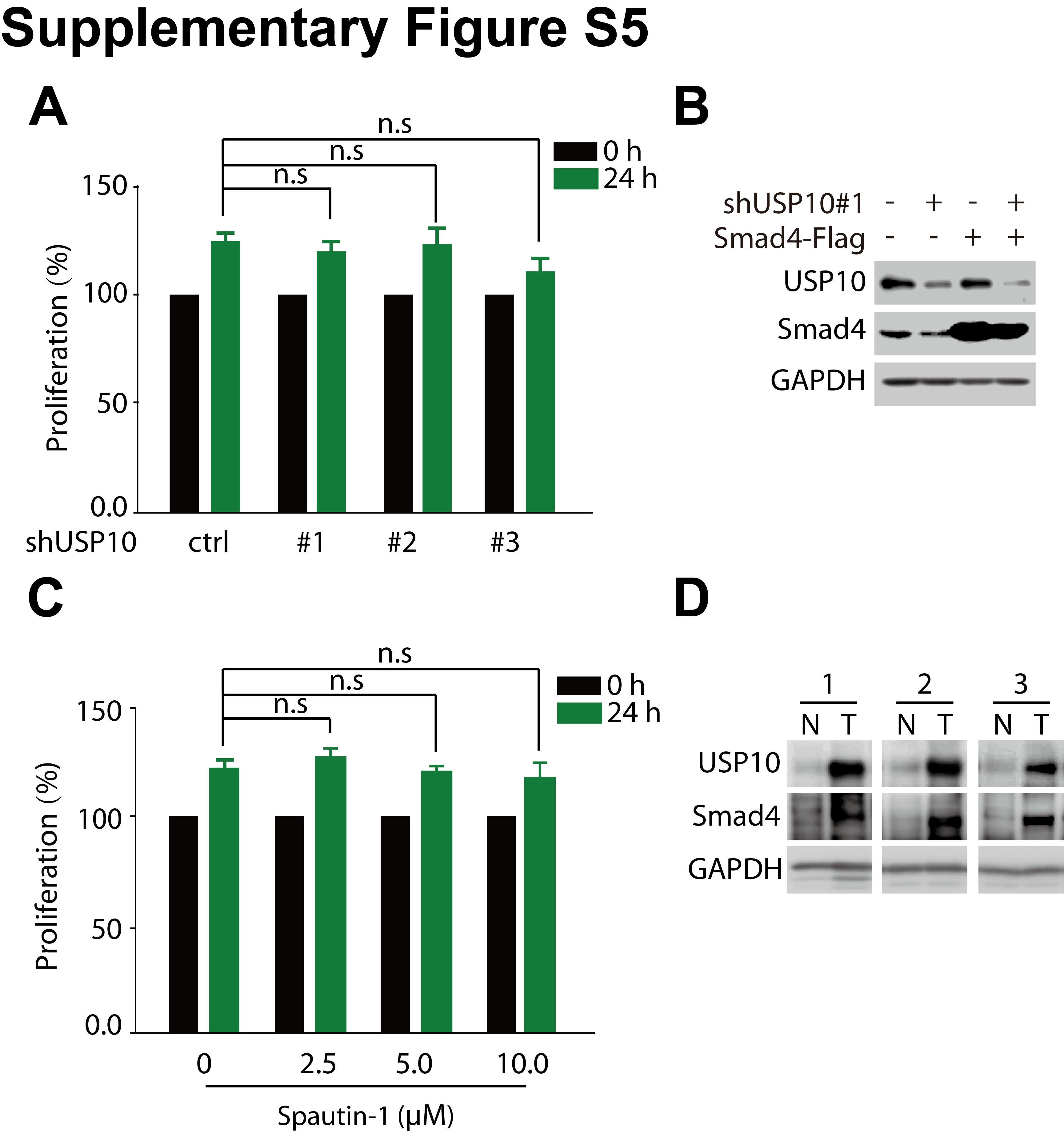

Supplement: Supplementary file 5 — Fig. S5. Depletion of USP10 or depriving of its catalytic activity with small molecule inhibitor Spautin‐1 imposed minimal effect on the cell proliferation. [file MOL2-14-197-s005.tif]
