# Supplementary material for: Deubiquitinating enzyme USP10 promotes hepatocellular carcinoma metastasis through deubiquitinating and stabilizing Smad4 protein
Source: Mol Oncol. 2019 Nov 27;14(1):197–210. doi: 10.1002/1878-0261.12596 (PMC6944132; doi:10.1002/1878-0261.12596)
Supplement: Supplementary file 6 — Table S1. Complete list of the siRNAs targeting 54 human known or predicted ubiquitin‐specific proteases (USPs). Table S2. The primer sequences for qRT‐PCR assay. Table S3. The targeting sequences for shRNA. [file MOL2-14-197-s006.pdf]

Supplementary Figure and Supplementary Figure Legends

Supplementary Figure S1. Positive correlation between Smad4 protein levels and HCC progression

(A) The survival rate significantly decreases in HCC patients with high levels of Smad4 and TGF-β compared to those with low expression levels (TCGA database). (B) The mRNA levels of Smad4 closely correlate with the high risk of HCC patients (TCGA database). (C) The mRNA levels of *SERPINE1/ JUNB/ CDKN2B* (three well-known TGF-β signaling target genes) significant associated with high risk of HCC patients (TCGA database). ***, $P < 0.001$.

Supplementary Figure S2. Positive correlation between USP10 protein and HCC progression

(A) The mRNA levels of USP10 closely correlate with the high risk of HCC patients (TCGA database). (B) The Progression-Free Survival (PFS) rate significantly decreases in hepatitis virus-related HCC patients with high levels of USP10 compared to those with low expression levels (Kaplan-Meier Plotter). (C) A positive correlation of gene expression correlation analysis between *USP10* and *CDKN2B* (GEPIA database). ***, $P < 0.001$.

Supplementary Figure S3. USP10 regulates Smad4 protein levels through the ubiquitin-proteasome system

(A) The semi-quantitative analyses using density measurements on the protein levels of Smad4 in Fig. 2A. (B) The effect of USP10 silencing on Smad4 can be restored by the proteasome inhibitor MG132. (C&D) The semi-quantitative analyses using density measurements on the protein levels of Smad4 in Fig. 2C (C) and Fig. 2E (D). (E) Spautin-1 imposed minimal effect on Smad4 protein levels in USP10-depleted cells. (F) Overexpression of USP10-WT/C424A had little effect on Smad4 mRNA levels. (G) Depletion of USP10 imposed little effect on the half-life of Smad2/3 protein. The results represent the means (±SD) of three independent experiments. n.s, $P > 0.05$, **, $P < 0.~01$, ***, $P < 0.001$.

Supplementary Figure S4. The types of poly-ubiquitination occurring on Smad4

(A&B) The semi-quantitative analyses using density measurements on the protein levels of smears of ubiquitin in Fig. 4A (A) and Fig. 4B (B). (C) The types of poly-ubiquitination occurring on Smad4. (D-F) The semi-quantitative analyses using density measurements on the protein levels of smears of ubiquitin in Fig. 4C (D), Fig. 4D (E) and Fig. 4E (F).

Supplementary Figure S5. Depletion of USP10 or depriving of its catalytic activity with small molecule inhibitor Spautin-1 imposed minimal effect on the cell proliferation

(A) Depletion of USP10 imposed minimal effect on the cell proliferation. (B) Western-blot to test the protein levels of Smad4 in Fig 5D and 5E. (C) Spautin-1 imposed minimal effect on the cell proliferation. (D) Highly correlation between USP10 and smad4 expression in tumor tissues. Western blot analysis of paired samples of non-tumorous tissues (N) and tumor (T) from the same DEN-induced HCC mice.

Supplementary Table S1. Complete list of the siRNAs targeting 54 human known or predicted ubiquitin-specific proteases (USPs)

Gene name	Sense siRNA Sequence (5'-3')
USP1	GCAUAGAGAUGGACAGUAU
	GAAAUACACAGCCAAGUAA
	CAUAGUGGCAUUACAAUUA
	GCACAAAGCCAACUAACGA
USP2	CCAGCAAGCUCACAACAUU
	UCGCUGACGUGUACAGAUU
	GCCGACAGAUGUGGAGAAA
	GGUUACUGUUCUACGGUCU
USP3	GAAGUAAGCGCUCUAAGAA
	GUAGAAGAGUUUAGAAAGA
	GCACACAGUAUGUAUGGAU
	CCUCAUAUGUGGGACAGAA
USP4	CCAAAUGGAUGAAGGUUUA
	GGAAUAAACUACUAAACUG
	GAUCUGAACCGGGUAAAGA
	GAAUUGGACUAUGUAUUGG
USP5	GAGCUGACGUGUACUCAUA
	GGACAACCCUGCUCGAAUC
	GGAGAGACAUUUCAAUAAG
	GAUCUACAAUGACCAGAAA
USP6	CAACGGACCUGGAUAUAGG
	GCGGAGAGGUUCACAACAA
	GAGCGGAAGGACAUACUUA
	GAACCUGAUUGACGGGAUC
USP7	CUAAGGACCCUGCAAAUUA

	GUGGUUACGUUAUCAAAUA
	UGACGUGUCUCUUGAUAAA
	GAAGGUACUUUAAGAGAUC
USP8	UGAAAUACGUGACUGUUUA
	GGACAGGACAGUAUAGAUA
	AAAUAAAGCUCAACGAGAA
	GGCAAGCCAUUUAAGAUUA
USP9X	CAAAGGAGAUUUACUAGAA
	AGAAAUCGCUGGUAUAAAU
	ACACGAUGCUUUAGAAUUU
	GUACGACGAUGUAUUCUCA
USP9Y	GGAAUGAAAUGCUUUGAAA
	GUACGGCGAUGUAUUGUUA
	GAAUGUACCUGCUACCUUU
	GCAGUUGUCCUGUUGCUUA
USP10	CCAUAAAGAUUGCAGAGUU
	CAAACAAGAGGUUGAGAUA
	CCACAUAUAUUUACAGACU
	GAGUUGCACACCACGGAAA
USP11	GGGCAAAUCUCACACUGUU
	GAACAAGGUUGGCCAUUUU
	GAUGAUAUCUUCGUCUAUG
	GAGAAGCACUGGUAUAAGC
USP12	CAUCAGAUAUCUCAAAGAA
	CCAGAUGUCUUACUUGUGA
	GAAACUCUGUGCAGUGAAU
	GAAGAAUUCUACGGGUUGA
USP13	GAAGAUGGGUGAUUUACAA

	GCACUGGAUUGGAUCUUUA
	GCACGAAACUGAAGCCAAU
	UGAUUGAGAUGGAGAAUAA
USP14	GCAUAUCGCUUACGUUCUA
	GGACUUAAAUUGCGACUUC
	CAAGAUGAAUGGAUUAAGU
	GGAGUUACCAUGUGGAUUG
USP15	GAAGAAGGCUCACCAAGUG
	GAACGCACCUUGGAAGUUU
	GCAGAUAAGAUGAUAGUUA
	CAGAUAAGGUGGUUGCCGA
USP16	CGAAUAAACUGCUUUGUGA
	GGAACAAGGUAAUUUGAAA
	GAACACAGUGGUACUAUGA
	CAAAUAGUCAUCUCUCUAA
USP17L2	GGAAAUACCUGCUACGAGA
	GCAGGUAGAUCAUCACUCU
	CCAAGAAUGUGCAAUAUCC
	CGGGAGCACUCUCAAACAU
USP17L9P	UGUCACAGGCAACAAGAUU
	GGGCUCUGCUUGUGUGCCA
	UCAAAAUACAAGUGUGGGA
	GCAGGUAGAUCAUCACUCU
USP18	GGAAGAAGACAGCAACAUG
	CGUUUGGGCUCCUGAGGCA
	UGAUUCAGGUGUUCGUAAU
	GAGCAGAGGAGAAGCGUCC
USP19	GAUGAGGAAUGACUCUUUC

	GAGGACACCACUAGUAAGA
	UGGCGGAGGUAAUUAAGAA
	UCAAGAAUGACUCGUAUGA
USP20	GCGAGUGGCUCAACAAGUU
	CAAGAAAGCCCAGGUAUUG
	UCUGAAAGCUGUUCCUAUU
	GAACGCCGAGGGCUACGUA
USP21	GUACAAAGAUUCCCUCGAA
	GAACCUGAGUUAAGUGAUG
	GAGCUGUCUUCCAGAAAUA
	GAGCAGCACUCGACCUCUU
USP22	GGAGAAAGAUCACCUCGAA
	CAAAGCAGCUCACUAUGAA
	GGAAGAUCACCACGUAUGU
	CCUUUAGUCUCAAGAGCGA
USP24	GGACGAGAAUUGAUAAAGA
	AGGGAAACCUUACCUGUUA
	CCACAGCUUUGUUGAAUGA
	GUAGAAGCCUUGUUGUUCA
USP25	GCAGAUGGAUGAAGUACAA
	UGAAAGGUGUCACAACAUA
	GAGCUGAGGUAUCUAUUUG
	CAAUUAAGUUGGAAUAUGC
USP26	CCACAAAGCUGGAGGUAAA
	GAAGAUACCUCACUUUGUC
	GCACAAGACUUCCGUUGGA
	CCACACAUUGGAUCAGAUA
USP27X	UCAUGUGCCCUAUAAGUUA

	GAUAUGACGCCGUUUAUGG
	GAUGUGAGAUGCCGAGUCC
	UAGCAGUAGACCUGUAUUA
USP28	GAAGGUGGCUCAAGCGAAA
	UGACACACAUAUGACAUUA
	CAACAAGGAAGUAUUAGCA
	UCAGGUGCCUUAUCGCUUG
USP29	GAAAGAAGCUCUCAUUGAA
	GAAUAUAACUGUCAGAUGU
	GUACAAAGAUCAAGAGAGA
	GAAGAACCCAUCAAGUUUA
USP30	ACAGGAUGCUCACGAAUUA
	GCCAAGAAGUUACUGAUGA
	GAGCAGCAGUCAGAAAUAA
	CCAGAGUCCUGUUCGAUUU
USP31	GAACCAAGCGACAGUCAUA
	GUAGACAGCUCUCCAGUCA
	CCUCAAACCUGCACUUUAU
	CAGCAUACAUCCUCUUCUA
USP32	GAACAACACCUGGUAAUUG
	GUAAAUGGUCGGUGGAUAA
	GCUUGGUGCCCAUGGUAUA
	CUGUCAAGUAGUAAAGAGA
USP33	GGAGAAGGAUAUCAAAUUU
	GACAGUGGCUUAAUAAAUU
	GAGAAGAUGUGCAAUAAGA
	GUAGUAACCUUGCAAGAUU
USP34	GAAAUUGACUCUCCUUAUU

	UAACAUGGCUGACUUAAUG
	GCAAUGAGGUUAAUUCUAG
	GGACCAAAUUUACAUAUUG
USP35	AGAGCGAGCUGGCGGGUUU
	GCUCGGAGUAUCUGAAGUA
	CAACAUCCUUUACCUACAG
	GGGCUUUGAUGAAGACAAG
USP36	GCACACCACUGAAGAGAUU
	GAAAGCAGAUGUCCUGAGU
	GGACUCGGCUGAUGAUGGA
	CCGUAUAUGUCCCAGAAUA
USP37	CAAAAGAGCUACCGAGUUA
	GCAUACACUUGCCCUGUUA
	AAACAAAGCCGCCUAAUGU
	GAGGAUCGAUUAAGACUGU
USP38	GGAAGUAGCUAGUAAAGCA
	GGAGACAAGUAUUAUCUUU
	GUCCAGAAGUUUACUAUUU
	GCAUAGUACUAAUGGUUUA
USP39	GAUCAUCGAUUCCUCAUUG
	CAAGUUGCCUCCAUAUCUA
	UCACUGAGAAGGAAUAUAA
	ACAUAAAGGCCAAUGAUUA
USP40	GAAACUAGCUGUUAUACAU
	GAAGAGAAAUGGGUCACUA
	GCAGAGAGUUGCCGAUUUC
	GAACGAGCCUGCGCAAGUU
USP41	GGAAGAAGACCCGUGGGAA

	CCAGGGAGUUAUCAAGCAA
	GGAAUUCACAGACGAGAAA
	GCGAGAGUCUUGUGAUGCU
USP42	UGACAAAGCUUCUGAAUCU
	UUAGAGACCUUCAGGCUUA
	CAACCUAACCUUCAUAGUA
	GAAUGGGAUUGGUACGAUU
USP43	GGGCUUAUAUCCUGUUCUA
	GUGAAAGGCAGAAGCAUUA
	GAAGAGGACCUGAAUACCA
	GUGCUAAUCCUCUUCUGUA
USP44	GAACAUGGUUUGAACAAUC
	GUAGAAAGAUGGAACUUAU
	GAAUUGGAGUAUCAAGUUA
	GAAGAUUGGUGUUCAUGUU
USP45	CCAGGAAAUUAUCGGAACA
	CUGUAAUUAAAGACGGUAA
	GAACAUAGUGGCUCGAUGA
	UGGCUUGAGUCUUCGUAAA
USP46	GAAACUCGAUGCUUGAACU
	CCAGAGCAGUUUCCAAUCA
	GGGAGAAUGUGUUGGCAUA
	UGAACGAACCUGCGGAAAA
USP47	GGACUUGACUCUCACAGUA
	CCUGAAAGCUGAAGGAUUU
	GAGAGAAGCUUAGUGAAAU
	GCAACGAUUUCUCCAAUGA
USP48	CUACAUCGCCCACGUGAAA

	GCACUCUACUUAUGUCCAA
	GGCAGAGAGUCUAAGCUUU
	CGAAUUGCUUGGUUGGUAU
USP49	CCAAACAGGUCUUAAAGGU
	GGAUAGAUGCAAACAUGUA
	UAUUAACCAUGGAACCUUA
	ACAAGGAGCCGAGUUCAAA
USP50	UAUGAUACCCUUCCAGUUA
	GAGAACGGAUAUUCAUUAC
	GGUUUGACAUUCAGGGUAC
	CAACACAUGCUGCGUGAAU
USP51	GAGCAGGGAUGACCACAUA
	AAACAAAGCAGCACCAUUU
	GCUUUAAAGUAGGUAAGAA
	GGUUGAUCUACCAGCGUUU
USP53	GAAGUUCCCUUGAAUCUAA
	GAAAGGACCAGCUAAGUUA
	GCAAUGAGGUUGAAAGAAU
	GAAAAGAACUCAGGAAUUU
USP54	GAUAGAAGUUUGUCAGGUA
	GAAUUUACGUCGCAUCUCA
	GGGAAGAGUCCACUGAACA
	GGUCACUGAUAGAGCGCAA
USPL1	CAAGAAAGCUCGUAAGAGU
	GGACACAGCUGUAACUCAU
	UCAAUUGAGUGGUGUUAAA
	GUAAUUCAGUAUCGAGCAA

Supplementary Table S2. The primer sequences for qRT-PCR assay

Genes	Forward (5' - 3')	Reverse (5' - 3')
Smad4	CTCATGTGATCTATGCCCGTC	AGGTGATACAACTCGTTCGTAGT
GAPDH	GGAGCGAGATCCCTCCAAAAT	GGCTGTTGTCATACTTCTCATGG
USP7	GATGAAAAGTCGTTCAGTCGTCG	TTTGAATCCCACGCAACTCCA
USP10	AAGCAAGCTATGGCTCCATCG	CTTCCGCCTCCACATTAGAAC
USP11	CGTTTCCGGGACCAGAATCC	CATCGCCGTCCGTTCTCTTC
USP21	CAGGTCTGCCTGATGAACGG	GCTAAGTTGGTCCGAGATGGG
USP26	TTGACACTTACTTGCGGAGAGT	ACACCTCGAACAATCTGCCTT
USP29	GAAACTCGGGCCTTCATTCAA	CTGTGCTCTGGTTCCAATGG
USP33	AAAATCCCTTGGTACTTGTCAGG	TCGAAGAGTGGTAAGGTTCACA
USPL1	GAGTATTGCCCTGCTTGTAGAG	CTGAGGCTTATGTGGAGTGTG

Supplementary Table S3. The targeting sequences for shRNA

shRNAs	Targeting Sequences (5'- 3')
shUSP7	TGTATCTATTGACTGCCCTTT
shUSP10#1	GCCTCTCTTTAGTGGCTCTTT
shUSP10#2	CCTATGTGGAAACTAAGTATT
shUSP10#3	GCTGTGGATAAACTACCTGAT
shUSP11	CCCTCCCTTCTAGTCTTTATT
shUSP21	GACCCTCTGCAATATCACTTT
shUSP26	CTACTTTCAATCCCATCGTTT
shUSP29	CCACTTTAGAGATAGGGCAAT
shUSP33	CCTGGCCCTATTTCAAATAAT
shUSPL1	CCTACCTCATTTCGATGAATA
shSmad4	GCAGACAGAAACTGGATTAAA
